# Supplementary material for: Attitudes and Barriers of Polish Women towards Breastfeeding—Descriptive Cross-Sectional On-Line Survey
Source: Healthcare (Basel). 2024 Sep 2;12(17):1744. doi: 10.3390/healthcare12171744 (PMC11394689; doi:10.3390/healthcare12171744)
Supplement: Supplementary file 1 [file healthcare-12-01744-s001.zip › healthcare-3134549-supplementary.pdf]

Dear Mother,

In the Department of Nutrition and Epidemiology, Medical University of Lodz, we conduct a study on the behaviors and attitudes of polish women towards breastfeeding. We believe that given answers will contribute to greater interest in barriers of exclusive breastfeeding. You must be 18 years or older to participate in the study.

Please to provide honest answers ((by selecting the one answer that you consider the best, unless the question states otherwise). We ensure that the survey is completely anonymous. Submission of a completed questionnaire means that you consent to the use of the information contained solely for scientific purposes (Act on the Protection of Personal Data of 29 August 1997, Journal of Laws No. 133, item 883). The information collected will be developed in the form of aggregated statistics.

Thank you for participating in the study*.*

Anna Garus-Pakowska, PhD

At the beginning we will ask you to mark the answer regarding participation in the study. Select the appropriate answer.

- Yes, I agree to participate in the study, I am over 18 years old and confirm completing the survey

- No, I do not want to participate in this study, I am not yet 18 years old and I am not completing the survey.

Remember that you can stop filling out the survey at any time and leave the study. If you have any questions, we are available: anna.garus-pakowska@umed.lodz.pl

Questionnaire Dep Nutr and Epidemiol

Sociodemographic questions

Age (years):

a) 18<25
b) 25<30
c) 30<40
d) >40

Age of birth of first child (years):

a) 18<25
b) 25<30
c) 30<40
d) >40

How many children do you have?:

a) 1
b) 2
c) 3
d) 4 and more

Your education

a) Lower secondary and vocational
b) Secondary general
c) University

Place of residence:

a) City > 100.000 inhabitants
b) City < 100.000 inhabitants
c) Rural

Method of termination of pregnancy:

a) Naturally (full-term pregnancy)
b) Naturally (premature birth)
c) Cesarean section (full-term pregnancy)
d) Caesarean section (necessary earlier termination of pregnancy)

Did a cesarean section or premature birth affect how you feed your baby?

a) Yes, I had problems, but with help I managed to overcome them and was able to breastfeed
b) Yes, unfortunately I had to bottle feed
c) No, I didn't have any problems
d) Not applicable

Were you pregnant with multiples?

a) Yes
b) No

If yes, did the multiple pregnancy affect the way you fed your children? (you can choose multiple answers)

a) Yes, there were problems with lactation
b) Yes, I had to switch to a different method of feeding because the children were not eating enough
c) Yes, I was unable to feed the children (organizational reasons rather than physiological ones related to lactation)
d) No, I had no problems

Questionnaire Dep Nutr and Epidemiol

Questionnaire

How did you want to feed your baby (before he was born)?

a) Breastfeeding
b) Breast milk from a bottle
c) Formula feeding
d) Mixed feeding

How do/did you feed your child?

a) Breastfeeding
b) Breast milk from a bottle
c) Formula milk
d) Mixing

If you have switched from breastfeeding to formula feeding, do you feel resentful towards yourself for not being able to breastfeed for various reasons?

a) Yes
b) No

Do you think you have enough knowledge about breastfeeding?

a) Yes
b) No
c) I don't know

Please mark on the scale below how you rate your level of knowledge about infant nutrition?

I have no knowledge – 1 – 2 – 3 – 4 – 5 - Very good knowledge

Do you know breastfeeding techniques/positions?

a) Yes
b) No

Do you think that formula milk is as valuable as mother's milk?

a) Yes
b) No
c) I don't know

Do you think that children who eat mother's milk have better immunity than those who eat formula milk?

a) Yes
b) No

Questionnaire Dep Nutr and Epidemiol

What do you think are the most important benefits of breastfeeding? (you can choose more than one answer)

a) Closeness to your baby
b) A sense of security
c) Building immunity
d) Building a common bond
e) "easy and quick" access to food
f) Reducing the risk of breast cancer
g) Lower costs than formula milk
h) Other:

What would help mothers breastfeed longer? (you can choose more than one answer)

a) Support from a lactation consultant/midwife after leaving the hospital
b) Reimbursement for lactation advice
c) Easy access to lactation advice
d) Family support
e) Increased knowledge about the benefits of breastfeeding
f) Knowledge of products that support lactation

Did you receive sufficient help in the hospital after giving birth to help you learn to breastfeed?

a) Yes
b) No

Did you seek help from a lactation consultant after you left the hospital?

a) Yes
b) No

Is it easy to get lactation advice in your area?

a) Yes
b) No
c) I don’t know

Does breastfeeding make it difficult to return to work?

a) Yes, it definitely makes it more difficult
b) Yes, rather yes, but it can be reconciled somehow (e.g. partner support, change of working hours)
c) No, it does not hinder

Did you return to work during breastfeeding (in the first year of the baby's life)?

a) Yes, my employer allowed me to take breaks to breastfeed
b) Yes, but I work from home
c) Yes, I switched to breastfeeding differently

Questionnaire Dep Nutr and Epidemiol

d) No, I breastfed for a year and only then returned to work
e) No, I did not return to work
f) No, I do not work professionally
g) Unfortunately, I had to return to work and therefore stop breastfeeding
h) Other.....

What do you think a woman who has trouble breastfeeding should do?

a) Consult a certified lactation consultant
b) Switch to formula milk and stop breastfeeding
c) Consult a doctor
d) Other:…………….

In your opinion, what are the most common reasons for an infant to reject the breast?

a) Using a pacifier
b) Bottle feeding
c) Formula feeding
d) Teething
e) Poor mother's diet
f) Expanding the infant's diet
g) Other:

According to you, the most important factors that reduce the production of breast milk are:

a) Stress
b) Lack of knowledge
c) Lack of support
d) poor diet
e) low-quality food
f) Other:…………………

Have you ever felt pressure from family/society to breastfeed?

a) Yes
b) No

Have you ever felt embarrassed to breastfeed in public or decided not to breastfeed your baby in such a place?

a) Yes
b) No

What do you think could be a barrier to breastfeeding a child in a public place? (you can choose more than one answer)

a) Being ashamed
b) Feeling naked

Questionnaire Dep Nutr and Epidemiol

c) Fear of the gaze of others
d) Lack of designated places
e) Feeling that society does not accept breastfeeding in the presence of others
f) Other:………….

Questionnaire Dep Nutr and Epidemiol
